# IgD型复发难治多发性骨髓瘤患者BCMA靶点再治疗1例报告并文献复习

**DOI:** 10.3760/cma.j.cn121090-20240426-00165

**Published:** 2024-10

**Authors:** 思楠 顾, 琬婷 强, 静 卢, 中原 冯, 鹃 杜

**Affiliations:** 海军军医大学第二附属医院（上海长征医院）血液病科，全军骨髓瘤与淋巴瘤疾病中心，上海 200003 Department of Hematology, The Myeloma & Lymphoma Center, The Second Affiliated Hospital of Naval Medical University (Shanghai Changzheng Hospital), Shanghai 200003, China

## Abstract

多发性骨髓瘤（MM）是一种恶性浆细胞疾病，目前仍无法治愈。近年来，新药不断问世，与传统治疗相比，以B细胞成熟抗原（BCMA）为靶点的新药极大地改善了MM的疗效及预后。本文报道1例BCMA×CD3双特异性抗体治疗失败的IgD型复发难治MM患者，经过七线治疗，再次接受基于BCMA靶点的CAR-T细胞治疗后获得深度缓解，并进行相关文献复习，以期提高临床医师对复发难治MM患者BCMA靶点再治疗的认识。

## Abstract (en)

Multiple myeloma（MM）is a malignant plasma cell disease that currently cannot be cured. Several new drugs have continuously been introduced in the recent years. New drugs targeting B-cell maturation antigen（BCMA）have greatly improved the efficacy and prognosis of MM compared with traditional treatments. This article reports the case of an IgD type relapsed and refractory MM patient with poor efficacy of BCMA×CD3 bispecific antibody. The patient achieved deep remission after receiving BCMA-targeted CAR-T cell therapy after initial seven lines of treatment. Literature review was also conducted to improve the clinical physicians' understanding of BCMA target therapy for relapsed and refractory MM patients.

多发性骨髓瘤（MM）是一种浆细胞恶性增殖性疾病，尽管治疗方式持续更新，该病至今仍无法治愈。BCMA主要在B系细胞中表达，是目前MM靶向治疗的研究热点。目前针对BCMA靶点的药物主要包括以艾基维仑赛（Idecabtagene vicleucel，Ide-cel）、西达基奥仑赛（Ciltacabtagene autoleucel，Cilta-cel）为代表的嵌合抗原受体T（CAR-T）细胞免疫疗法，以玛贝兰妥单抗（Belantamab Mafodotin）为代表的抗体药物偶联（antibody-drug conjugate，ADC）类药物和以Teclistamab（Tec）及Elranatamab为代表的BCMA双特异性抗体（双抗）类药物。上述药物靶点类似但药理机制不同，耐药后是否可以互相更换、不同种类药物选择的先后顺序目前尚有争论。本文报道1例BCMA×CD3双抗治疗失败的IgD型复发难治MM，经过七线治疗，再次接受基于BCMA靶点的CAR-T细胞治疗，取得深度缓解，并结合文献进行复习，以期提高临床医师对复发难治MM患者BCMA靶点再治疗的认识，为复发难治MM患者的治疗方案提供参考，同时提高对罕见IgD型MM的诊疗认知。

## 病例资料

患者，女性，54岁。2019年7月因腰背部反复疼痛入院，胸、腰椎CT、腰椎正侧位片均提示L1椎体病理性骨折，腰椎CT提示多发骨质破坏。于2019年8月于外院行经皮椎体成形术，术后仍感疼痛，性质同前。2019年10月于手术医院复诊时实验室检查示HGB 80 g/L，乳酸脱氢酶331 U/L，β_2_-微球蛋白4.31 mg/L。骨髓穿刺涂片见骨髓瘤细胞占14％，血清蛋白电泳、免疫固定电泳均未见异常，血清游离轻链结果不详。外院诊断为MM，λ轻链型，DS分期ⅢA期。2019年10月至2020年4月予VAD（硼替佐米+表阿霉素+地塞米松）方案化疗5个疗程，后因手足麻木、刺痛等周围神经病变，调整为IAD方案（伊沙佐米+表阿霉素+地塞米松）化疗1个疗程。2020年7月行自体造血干细胞采集，8月复查评估，移植前疗效达到完全缓解（CR），行自体造血干细胞移植。2020年12月起口服伊沙佐米维持治疗，1个月后因手足麻木更换为来那度胺维持治疗，1个疗程后停用全部治疗MM的药物，定期随访，病情稳定。

2021年8月患者于当地医院随访时发现血常规异常（具体不详），2021年9月骨髓涂片示浆细胞占15％，流式细胞术示7.63％异常浆细胞，提示疾病第1次复发。2021年9月患者至我院就诊，骨髓瘤相关实验室检查示：血M蛋白IgD-λ型，定量5.849 3 g/L；血清IgD>16 640 mg/L；血清游离轻链（sFLC）：κ轻链10.93 mg/L，λ轻链292.37 mg/L，κ/λ 0.037 4。骨髓涂片：浆细胞占59％。FISH：1q21扩增占23％。诊断为MM，IgD-λ型，DS分期ⅢA期，ISS分期Ⅲ期，R-ISS分期Ⅲ期。2021年9月起予PCD（泊马度胺+环磷酰胺+地塞米松）方案治疗。2021年10月复查评估1个疗程血液学疗效为疾病稳定（SD），2021年10月起调整为DPD（达雷妥尤单抗+泊马度胺+地塞米松）方案治疗，至2022年9月共予DPD方案治疗8个疗程，最佳疗效为严格意义的CR（sCR），骨髓微小残留病（MRD）（+）。

2022年8月患者自觉前胸壁出现约1 cm×1 cm包块，进行性增大，质硬，活动度差。2022年9月复查血清蛋白电泳及免疫固定电泳均未见异常；sFLC：κ轻链8.47 mg/L，λ轻链113.12 mg/L，κ/λ 0.074 9。血清IgD 2 175.9 mg/L。骨髓穿刺涂片：浆细胞47.5％。患者入组Tec（人源BCMA×CD3双特异性抗体）Ⅰ/Ⅱ期临床试验（临床试验注册号：CTR20212614），2022年9月29日、2022年10月1日予启动剂量，2022年10月4日、2022年11月3日行2个疗程治疗。患者胸骨前肿块疼痛加重且影像学评估病变体积较前增大1倍，评估为疾病进展，2022年11月12日出组。2022年11月22日至2023年6月28日予SKD（塞利尼索+卡非佐米+地塞米松）方案治疗3个疗程。2023年7月初再次出现骨痛加重，血清IgD进行性升高，在SKD方案基础上加用苯达莫司汀及沙利度胺。2023年7月30日原发病评估：sFLC：κ轻链1.15 mg/L，λ轻链74.86 mg/L，κ/λ 0.015 4。血清IgD 2 411.7 mg/L。骨髓穿刺涂片：浆细胞占37％。骨髓流式细胞术见单克隆浆细胞28.46％，BCMA（+）细胞占81.03％。PET-CT显示，除全身骨质破坏伴代谢增高外，尚见右侧胸膜不规则增厚伴代谢增高，右侧胸腔积液伴右肺部分不张，考虑为MM髓外浸润，恶性胸腔积液。患者已经过七线治疗，处于疾病终末阶段，患者及家属强烈要求进行CAR-T细胞治疗，故予BCMA CAR-T细胞药物伊基奥仑赛注射液治疗。

患者于2023年8月行首次淋巴细胞采集，采集前PET-CT提示颅骨、双侧肱骨上段、胸廓、脊柱、骨盆、双侧股骨及胫骨多发骨质破坏，SUVmax＝4.66。淋巴细胞采集术后予SKTD（塞利尼索+卡非佐米+沙利度胺+地塞米松）方案治疗，但因淋巴细胞活力差，首次制备失败。2023年8月24日再次行淋巴细胞采集，淋巴细胞采集后行SKTCD（塞利尼索+卡非佐米+沙利度胺+环磷酰胺+地塞米松）方案治疗。患者于2023年9月入院行CAR-T细胞治疗，回输前相关指标：血M蛋白IgD-λ型，M蛋白定量为0.504 g/L。血清IgD 4 680.8 mg/L，sFLC：κ轻链<0.54 mg/L，λ轻链98.30 mg/L，κ/λ 0.005 5。为明确胸腔积液性质行胸腔积液引流，胸水测IgD 4 309.8 mg/L，游离轻链：κ轻链0.66 mg/L，λ轻链183.06 mg/L，κ/λ 0.003 6。胸水流式细胞术见65.59％的单克隆浆细胞，明确为MM胸膜浸润。2023年9月16日予FC方案预处理，9月20日行CAR-T细胞回输，回输剂量6.0×10^6^/kg。治疗过程中出现Ⅳ级中性粒细胞减少，Ⅳ级血小板减少。回输后7 d起CAR-T细胞拷贝数开始扩增，回输后8 d起出现Ⅰ级细胞因子释放综合征（CRS），持续2 d，予托珠单抗治疗CRS后症状缓解。无免疫效应细胞相关神经毒性综合征（ICANS）、巨噬细胞活化综合征（MAS）及其他严重治疗相关不良反应。治疗期间患者经流式细胞术测定CAR-T细胞绝对值最高为788/µl，IL-6最高为>2 500 pg/ml。CAR-T细胞回输后14 d查sFLC降至正常，回输后20 d查血清IgD 1 315.17 mg/L，胸水流式细胞术示单克隆浆细胞降至0.05％，根据本中心提出的针对IgD型MM的疗效评估体系[1]，评估疗效为部分缓解（PR）。回输后28 d查血清蛋白电泳阴性，免疫固定电泳IgD型，血清IgD 382.6 mg/L，sFLC：κ轻链<0.58 mg/L，λ轻链<1.24 mg/L，骨髓涂片浆细胞占0.5％，MRD未见单克隆浆细胞残留（经二代流式细胞术测定，阳性阈值为1×10^−6^），总体疗效评估为非常好的部分缓解（VGPR），MRD（−）。2023年12月查血清蛋白电泳及免疫固定电泳均阴性，sFLC：κ轻链<0.58 mg/L，λ轻链<1.24 mg/L，胸部CT均提示多发骨质破坏，较前相似。回输后3个月评估血液学疗效为sCR，MRD（−）。患者的治疗过程及疗效见表1。

**表1:** 患者治疗过程及疗效

线数	治疗方案	最佳疗效	PFS期（月）
1	VAD	CR	–（因不良反应换药）
2	IAD、auto-HSCT、伊沙佐米	CR	23
3	PCD	SD	1
4	DPD	sCR，MRD（+）	11
5	Teclistamab	SD	2
6	SKD	sCR	7
7	SKTD、苯达莫司汀	PD	<1
8	抗BCMA CAR-T细胞	sCR，MRD（−）	–

**注** VAD：硼替佐米+表阿霉素+地塞米松；IAD：伊沙佐米+表阿霉素+地塞米松；PCD：泊马度胺+环磷酰胺+地塞米松；DPD：达雷妥尤单抗+泊马度胺+地塞米松；SKD：塞利尼索+卡非佐米+地塞米松；SKTD：塞利尼索+卡非佐米+沙利度胺+地塞米松；BCMA CAR-T细胞：靶向B细胞成熟抗原的嵌合抗原受体T细胞；CR：完全缓解；SD：疾病稳定；sCR：严格意义的完全缓解；MRD：微小残留病；PD：疾病进展；PFS：无进展生存；–：无数据

## 讨论及文献复习

该患者因腰背部疼痛起病，外院诊断为λ轻链型MM后至我院明确诊断为IgD-λ型MM。IgD型MM是MM中相对罕见的亚型，在全球MM患者中占1％～2％[2]，亚洲患者中占比更高，为2％～8.8％[3]。通常被认为较其他亚型预后更差，在传统化疗时代的中位总生存（OS）期仅有9～21个月，随着新药时代的来临，IgD型MM的中位OS期明显延长至46～51.5个月[4]，但短于其他亚型MM（57～61个月）。近期一项研究统计了英国2003–2016年参加Ⅲ期临床试验的70例IgD型MM患者的临床特征和结局，发现与其他亚型相比，IgD型MM患者M蛋白水平较低（<10 g/L）[5]，使IgD型MM更难被早期识别与诊断，进一步影响了该亚型MM的预后[6]。本中心牵头进行了一项回顾性临床研究，纳入亚洲3个国家14个中心256例IgD型MM患者，是目前关于IgD型MM的最大宗报道，基于多变量Lasso回归分析建立了一项纳入5个临床特征的预后模型，以对IgD型MM患者进行更有效的风险分层[3]。该患者病程中M蛋白及血清游离λ轻链一直处于较低水平，末次进展时M蛋白及sFLC根据IMWG疗效评估标准均属于不可测量病灶，由此可见将血清IgD定量纳入疗效评估标准的重要性。本中心自2013年起建立IgD定量检测平台，将血清IgD浓度与IMWG疗效评估标准相结合，优化了IgD型MM患者的疗效评估系统，得以更早发现此类患者的疾病进展状态[1]。目前以IgD定量监测作为疗效评估手段的文献报道极少，亟待加强对相关指标的关注。

本例患者在含蛋白酶体抑制剂、免疫调节剂、CD38单抗方案治疗进展后入组Tec临床试验作为第五线治疗，但在1.5个月（2个疗程）后出现髓外病灶进展。Tec是一种T细胞重定向双特异性抗体，可以同时靶向T细胞表面表达的CD3和浆细胞表面表达的BCMA。Tec首先与T细胞表面的CD3结合，激活T细胞并使其能够识别和攻击肿瘤细胞，后与MM细胞表面的BCMA结合，将T细胞引导到肿瘤细胞附近，从而增强T细胞对肿瘤细胞的杀伤作用。针对Tec的Ⅰ/Ⅱ期临床试验（MajesTEC-1）共纳入165例复发难治MM患者，总体缓解率（ORR）为63.0％，中位反应持续时间为18.4个月，中位无进展生存（PFS）期为11.3个月。R-ISS Ⅲ期、髓外浆细胞瘤和骨髓浆细胞≥60％的患者应答率通常较低[7]，但高危遗传学患者对Tec的总体反应率与标危细胞遗传学患者相似[8]。MM对双抗治疗耐药的机制可分为免疫相关因素及肿瘤相关因素，免疫相关因素包括效应T细胞耗竭及骨髓内免疫抑制微环境形成，双抗治疗的效果取决于免疫效应细胞，患者的T细胞群在免疫抑制性肿瘤微环境中发生以终末分化、效应功能丧失和抑制性受体表达为特征的耗竭，对骨髓和外周血T细胞行单细胞测序显示，CD8^+^TOX^+^T细胞的耗竭与治疗失败有关[9]，双抗治疗过程中对效应T细胞反复刺激可能加剧T细胞的耗竭，同时，骨髓中单克隆浆细胞与骨髓微环境中其他辅助细胞发生复杂的相互作用，共同促进MM细胞的存活和生长，同时抑制有效的抗肿瘤免疫活性。肿瘤相关因素则包括肿瘤细胞抗原丢失、高肿瘤负荷及高可溶性BCMA水平。该患者为IgD型MM，合并髓外侵犯，且自身淋巴细胞活力减弱，可能是患者对双抗治疗不敏感、早期进展的原因。

基于已发表的小样本临床观察，经其他BCMA靶向治疗失败的患者仍可从抗BCMA CAR-T细胞治疗中获益，ORR虽低于既往未暴露于BCMA靶向治疗的患者，但较其他BCMA靶向治疗失败后再次接受BCMA双抗治疗的患者更高（ide-cel：74％[10]，Cilta-cel：60％[11]，Teclistamab：45％[12]，Elranatamab：54％[13]）。但研究亦表明，尽管BCMA双抗治疗失败患者经抗BCMA CAR-T细胞治疗获得了较好的反应率，但疗效和预后的亚组分析显示，与未暴露于BCMA靶向疗法的亚组和既往接受ADC/CAR-T细胞疗法的亚组相比，既往双抗治疗失败患者的缓解持续时间、PFS及OS更差[10],[14]。从机制上分析，CAR-T细胞治疗失败的患者肿瘤组织通常仍表达BCMA[15]–[16]，其治疗失败的原因常为CAR-T细胞耗竭[17]，而非BCMA等位基因突变导致的免疫逃逸，因此CAR-T细胞治疗后复发的患者可以从后续ADC或双抗治疗中获益。上述数据提示，在不考虑经济原因及药物可及性等因素的情况下，CAR-T细胞治疗可能应优先于靶向BCMA双抗/ADC药物治疗。在既往已接受过靶向BCMA双抗/ADC药物治疗的患者中观察到，药物诱导的T细胞耗竭可随无治疗间歇期的延长而改善[18]。既往接受BCMA靶点治疗后再次接受其他BCMA靶点治疗的相关研究总结见表2。简言之，既往靶向BCMA双抗/ADC药物接触少、停药时间久的患者对后续抗BCMA CAR-T细胞治疗的反应、疗效及预后更好。

**表2:** 既往接受BCMA靶点治疗后再次接受其他BCMA靶点治疗的相关研究

文献	既往抗BCMA治疗	研究应用抗BCMA治疗	例数	治疗反应	预后
Cohen等[11]	非抗BCMA CAR-T细胞	Clita-cel	20	ORR 60%，MRD阴性率35%	DOR：11.5个月；PFS期：9.1个月
Touzeau等[12]	抗BCMA CAR-T细胞	Teclistamab	11	ORR 45%	–
	BCMA靶向ADC药物	Teclistamab	16	ORR 38%	–
Raje等[13]	抗BCMA CAR-T细胞	Elranatamab	13	ORR 54%，≥VGPR率46%	–
Ferreri等[10]	BCMA靶向ADC药物	Ide-cel	37	ORR 68%，≥VGPR率46%	PFS期：3.2个月
	BCMA双抗	Ide-cel	7	ORR 86%，≥VGPR率43%	PFS期：2.8个月
	抗BCMA CAR-T细胞	Ide-cel	5	ORR 100%，≥VGPR率80%	中位PFS：未达到^a^
Mohan等[19]	BCMA靶点治疗	Teclistamab	38	ORR 54%	–
Riedhammer等[20]	Ide-cel	Teclistamab	21	ORR 33.3%，≥VGPR率21%	PFS期：1.8个月；中位DOR：未达到^b^
	BCMA靶向ADC药物	Teclistamab	23	ORR 73.9%，≥VGPR率65.2%	–

**注** BCMA：B细胞成熟抗原；CAR-T细胞：嵌合抗原受体T细胞；ADC：抗体药物偶联；Ide-cel：艾基维仑赛；Clita-cel：西达基奥仑赛；ORR：总体缓解率；MRD：微小残留病；VGPR：非常好的部分缓解；DOR：缓解持续时间；PFS：无进展生存；–：无数据；^a^中位随访时间4.5个月；^b^中位随访时间5.5个月

该患者在Tec治疗失败后尝试了以塞利尼索、卡非佐米、苯达莫司汀为基础的方案，但治疗过程中患者出现反复感染，且血清IgD持续升高，提示治疗反应不佳，于2023年9月行伊基奥仑赛（CT103A）治疗。伊基奥仑赛是全球首个全人源CAR-T细胞产品，以慢病毒为基因载体转染自体T细胞，共刺激域是4-1BB。伊基奥仑赛具有独特的全人源scFv，同时具有重链和轻链结合域，可较充分地覆盖人MM细胞BCMA抗原表位，有助于绕过宿主的抗CAR免疫原性，减少抗药物抗体等的影响，在免疫原性低的同时保留了抗肿瘤活性。在我国开展的一项Ⅰ期临床试验纳入接受≥3线治疗的复发难治MM患者，结果显示，该产品ORR为100％，72.2％的患者达到CR及以上疗效，既往接受CAR-T细胞治疗患者的sCR率为75％，随访12个月的PFS率为58.3％[21]。徐州医科大学附属医院的一项单臂、Ⅱ期临床试验报道，7例复发难治IgD型MM患者（既往接受2～9线治疗，中位接受4线治疗）接受抗BCMA CAR-T细胞治疗后，6例获得sCR，另外1例合并髓外病变的患者最佳疗效为微小缓解，评估骨髓MRD情况的6例患者均获得了MRD阴性[22]。该患者回输后28 d评估疗效为VGPR，MRD（−）。治疗过程中主要的治疗相关不良反应为Ⅳ级中性粒细胞减少、Ⅳ级血小板减少及Ⅰ级CRS，无ICANS、MAS及其他严重治疗相关不良反应。

综上所述，该患者为少见的IgD型MM，经免疫调节剂、蛋白酶体抑制剂、CD38单抗、BCMA×CD3双抗、XPO1抑制剂等多种新药治疗后反复进展，接受人源抗BCMA CAR-T细胞治疗后28 d即获得VGPR，MRD阴性。目前完成CAR-T细胞治疗后6个月，疗效为sCR，MRD（−），且治疗过程中非血液学不良反应仅有Ⅰ级CRS，治疗相关不良反应可控。患者既往抗BCMA治疗耐药，但CAR-T细胞治疗前患者的骨髓浆细胞高表达BCMA，BCMA（+）细胞所占比例为81.03％，因此接受抗BCMA CAR-T细胞治疗后仍能取得满意疗效。提示抗BCMA双抗治疗失败后，仍有部分患者可从抗BCMA CAR-T细胞治疗中获益，因这部分患者对双抗耐药的机制可能是患者自身T细胞耗竭，而非肿瘤细胞免疫逃逸。CAR-T细胞治疗前明确MM细胞的抗原表达情况可有助于对CAR-T细胞疗效的预测。另外，血清IgD定量对于IgD型MM患者的预后评估具有极大价值，临床上需注意对这部分患者的IgD水平进行动态监测。
